# Mesocarnivores as Sentinels of Environmental Metal(Loid) Exposure in a Mediterranean Protected Area

**DOI:** 10.3390/biology15212038

**Published:** 2026-11-08

**Authors:** Juan F. Osuna-Flores, Rafael Mora-Medina, Pablo Olmedo, Fernando Gil, Javier Vázquez-Rodríguez, Pedro J. Garrote-García, Antonio J. Lora-Benítez, Rafael Zafra Leva, Pablo J. Rufino-Moya, Mª Rosario Moyano-Salvago, Nahúm Ayala-Soldado

**Affiliations:** 1 Department of Comparative Anatomy and Pathological Anatomy and Toxicology, Competitive Research Unit on Zoonoses and Emerging Diseases from a One Health Perspective (ENZOEM), Faculty of Veterinary Medicine, Rabanales Campus, University of Córdoba, 14071 Córdoba, Spain; b42osflj@uco.es (J.F.O.-F.); v12lobea@uco.es (A.J.L.-B.); r.moyano@uco.es (M.R.M.-S.); 2 Department of Legal Medicine, Toxicology and Physical Anthropology, Faculty of Medicine, University of Granada, 18016 Granada, Spain; polmedopalma@ugr.es (P.O.); fgil@ugr.es (F.G.); 3 Asociación Lutra, 21200 Aracena, Spain; javiervazquezveterinario@gmail.com; 4 Department of Conservation Biology and Global Change, Doñana Biological Station (EBD-CSIC), 41092 Seville, Spain; pedro.garrote@csic.es; 5 Unidad de Parasitología (ParaCOR), Departamento de Sanidad Animal, UIC Zoonosis y Enfermedades Emergentes ENZOEM, Universidad de Córdoba, Ctra. Madrid-Cádiz, km 396, 14071 Córdoba, Spain; v62zafle@uco.es (R.Z.L.); v32rumop@uco.es (P.J.R.-M.)

**Keywords:** bioaccumulation, environmental monitoring, Mediterranean ecosystem, wildlife bioindicators

## Abstract

Metal(loid)s pose an ecological concern due to their persistence, toxicity, and potential for bioaccumulation in terrestrial ecosystems. In this study, concentrations of 14 elements, including As, Cd, and Pb, were determined in liver and kidney tissues from three mesocarnivore species collected as roadkill in and around Sierra de Aracena and Picos de Aroche Natural Park (SW Spain) between 2015 and 2018. Accumulation patterns differed among species and tissues. The Eurasian badger (*Meles meles*) showed the highest overall burdens, particularly for Cd in the kidney, consistent with greater long-term exposure. The stone marten (*Martes foina*) showed the lowest concentrations, whereas the Egyptian mongoose (*Herpestes ichneumon*) displayed intermediate values. Essential elements were generally more concentrated in liver than kidney. These findings indicate that wild mesocarnivores, especially Eurasian badgers, may be useful bioindicators of metal(loid) exposure; screening comparisons found no main Cd or Hg reference-value exceedances, although three badgers had renal Cd within an experimental effect range.

## Simple Summary

Wild animals can accumulate metals and related elements from their surroundings, even in protected areas. We compared exposure in three wild mammal species to assess their potential value for environmental monitoring. We measured 14 elements in liver and kidney samples from 41 Eurasian badgers, stone martens, and Egyptian mongooses found dead on roads in and around Sierra de Aracena and Picos de Aroche Natural Park, southwestern Spain. Accumulation differed among species and organs. Eurasian badgers generally showed the highest overall accumulation, particularly of cadmium in the kidneys, whereas stone martens showed the lowest. Egyptian mongooses had the highest mercury concentrations in the liver. Kidney cadmium and liver mercury concentrations remained below the main reference values used to screen for potential harm. However, three badgers had kidney cadmium concentrations within a range associated with kidney changes in laboratory mice. This finding warrants further investigation but does not demonstrate kidney damage in badgers. These results suggest that wild mammals, especially Eurasian badgers, can provide useful information for environmental monitoring. Studying animals already killed on roads offers a practical approach without additional wildlife capture and may help guide conservation efforts in protected landscapes.

## 1. Introduction

Metal(loid) exposure is a major environmental concern due to the persistence, toxicity, and bioaccumulative potential of many elements in terrestrial ecosystems. These elements originate from both natural and anthropogenic sources and, once released into the environment, may accumulate in soils and water, where their ecological impact depends largely on mobility and bioavailability [1]. They may subsequently enter plant and animal tissues and be transferred through food webs, thereby threatening biodiversity and compromising ecosystem integrity [2,3]. Studies of contaminated terrestrial ecosystems have also documented the transfer of potentially toxic elements among soil, plants, leaf litter, and earthworms [4].

In terrestrial ecosystems, mesocarnivores play important ecological roles as predators, scavengers, and regulators of prey populations. Because they occupy intermediate to high trophic levels, these species are considered useful bioindicators of environmental metal(loid) exposure [5,6,7]. Pollutant concentrations in their tissues may reflect both direct environmental exposure and dietary uptake through the food web, thereby providing valuable information on contaminant transfer and ecosystem health.

Sierra de Aracena and Picos de Aroche Natural Park, located in the province of Huelva (Andalusia, SW Spain), occupies the western sector of the Sierra Morena range and is recognized for its high ecological value and importance for the conservation of Mediterranean habitats and wildlife. The park includes characteristic ecosystems such as dehesas and oak and chestnut forests, which support rich biodiversity and provide important ecological functions [8,9].

Protected status does not preclude environmental metal(loid) exposure. In mineralised sectors of Sierra de Aracena, weathering of sulphide-bearing bedrock has produced soils naturally enriched in elements such as Zn, Pb, Cd and As [10]. In the wider Huelva region, elevated soil concentrations of As and Pb have also been documented in the mining area of Minas de Riotinto compared with Aracena [11]. These findings provide a regional rationale for investigating metal(loid) exposure in wildlife, while recognising that environmental concentrations alone do not establish uptake or tissue accumulation.

Previous studies quantified seven elements in the liver, muscle and bone of carnivores from Doñana and Sierra Morena [12], and five elements in the liver and kidney of Eurasian badgers and European hedgehogs from north-western Spain [13].

The availability of roadkill specimens allowed comparison of Eurasian badgers, stone martens and Egyptian mongooses, whose feeding habits may lead to different metal(loid) exposures. In Mediterranean woodlands, Eurasian badgers consume fruits and arthropods, with seasonal shifts in food use [14]. Stone martens combine fruits with animal prey [15], whereas Egyptian mongooses consume varied vertebrate and invertebrate prey according to availability [16]. Comparing these species can reveal exposure profiles that may be missed when monitoring a single species; dietary pathways remain hypotheses because diet was not measured in the sampled individuals.

Liver and kidney were selected to assess complementary patterns of element accumulation. Previous work in mesocarnivores identifies liver as an important storage organ for essential elements such as Cu, Fe, Mn and Zn, and kidney as a site of Cd accumulation [7]. Higher Cd concentrations in the kidney than in the liver have also been reported in Eurasian badgers from north-western Spain [13].

The present study aimed to compare the concentrations and accumulation patterns of 14 elements, including As, Cd and Pb, in the liver and kidney of Eurasian badgers, stone martens and Egyptian mongooses from Sierra de Aracena and Picos de Aroche Natural Park and its surroundings, and to assess potential toxicological risk using published tissue-residue reference values. Using a common analytical protocol, we combined multivariate analysis and the Metal Pollution Index (MPI) with a screening-level toxicological risk assessment. This comparison extends previous evidence and can inform the selection of species, organs and elements for future monitoring.

## 2. Materials and Methods

### 2.1. Sample and Location

**Figure 1:**
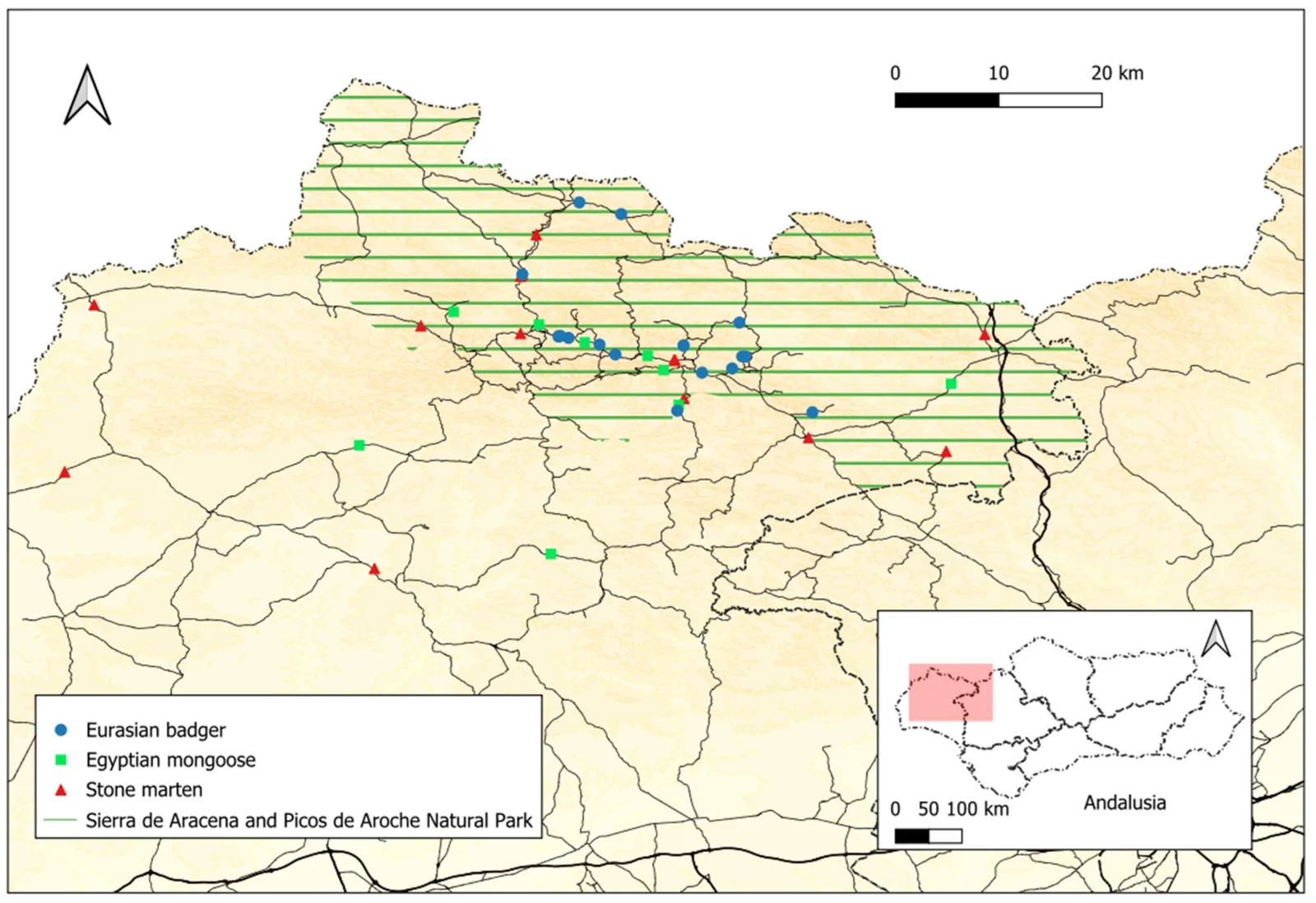
Locations where carcasses of the three mesocarnivore species were collected within Sierra de Aracena and Picos de Aroche Natural Park and surrounding areas. Black lines indicate the road network.

The study included 41 roadkill specimens (9 Egyptian mongooses, 14 stone martens and 18 Eurasian badgers), collected opportunistically in the study area between 2015 and 2018 (Figure 1). Sampling coverage was uneven across years and seasons. Sampling was not stratified by anthropogenic pressure or environmental contamination. Environmental metal(loid) concentrations were not measured at individual sites, so differences in contamination among locations were not quantified. Coordinates mark carcass recovery sites and should not be interpreted as precise locations of contaminant uptake.

Species identification was based on morphological characteristics, and scientific nomenclature followed Fauna Europaea [17]. Sex was determined for 35 of the 41 individuals by external examination of the genitalia and/or inspection of the reproductive tract during necropsy, when carcass condition permitted. The sample comprised seven females, six males and five individuals of undetermined sex among Eurasian badgers; eight females, five males and one of undetermined sex among stone martens; and six females and three males among Egyptian mongooses. Body weight was available for 32 individuals; age was not recorded. Individual sex and body-weight data are provided in the Supplementary Data.

The interval preceding carcass recovery varied with notification and collection circumstances. A qualified veterinarian performed necropsies as soon as practicable after recovery. Variable carcass preservation prevented collection of both organs from every individual, yielding 40 kidney and 33 liver samples. Kidney/liver sample numbers were 9/9 for Egyptian mongooses, 14/8 for stone martens and 17/16 for Eurasian badgers. Samples were sealed and kept frozen at −20 °C without further handling until analysis; storage duration varied among samples.

### 2.2. Metal(Loid) Determination

Approximately 0.7 g of each liver and kidney sample was weighed and digested in quartz vessels using 1 mL of HNO_3_ (Suprapur, Merck, Darmstadt, Germany) and 1 mL of ultrapure water (Milli-Q, Merck) in a microwave digestion system (ETHOS UP, Milestone, Sorisole, Italy). The analytical portion mass was consistent with that previously used for elemental analysis of animal tissues by Olmedo et al. [18]. Digestion was performed at a maximum power of 1800 W and a maximum temperature of 210 °C, with a 20 min ramp time, 15 min hold time, and 60 min cooling time. Following microwave digestion and cooling, the digestates were visually inspected for residual tissue and significant turbidity before dilution and ICP-MS analysis. After digestion, the samples were transferred to decontaminated tubes for subsequent analysis. Before use, quartz vessels were cleaned, soaked for 24 h in 10% HNO_3_(Sigma-Aldrich, St. Louis, MO, USA), thoroughly rinsed with ultrapure water, and dried at 80 °C for approximately 2 h.

Concentrations of 14 elements (As, Cd, Co, Cr, Cu, Fe, Hg, Mg, Mn, Mo, Ni, Pb, Se, and Zn) were determined in liver and kidney samples. Calibration was performed using multielement standards (Agilent Technologies, Santa Clara, CA, USA) prepared in a solution containing 2% (*w*/*v*) HNO_3_ (Suprapur, Merck, Darmstadt, Germany) and 1% (*w*/*v*) HCl (Suprapur, Merck, Darmstadt, Germany) in ultrapure water. All digested samples were diluted 1:10 in 1% (*w*/*v*) HCl prepared with ultrapure water prior to analysis. Procedural blanks were included to correct the results. Multielement determination was carried out by inductively coupled plasma mass spectrometry (ICP-MS) using an Agilent 8900 triple quadrupole ICP-MS instrument (Agilent Technologies, Santa Clara, CA, USA). Instrument tuning and performance checks were conducted before analysis. A solution containing 400 µg/L Sc, Ge, Ir, and Rh (ISC Science, Gijón, Spain) was introduced online as an internal standard to correct for signal drift and potential matrix effects.

Analytical performance was monitored by analysing the certified reference material ERM-BB185 (bovine liver; European Commission, Joint Research Centre, Geel, Belgium), together with a blank and an intermediate calibration standard, every 12 samples. In addition, NIST SRM 1640a Trace Elements in Natural Water (National Institute of Standards and Technology, Gaithersburg, MD, USA) was analysed at the beginning and end of each analytical batch. Recoveries for target elements in certified reference materials ranged between 83.0% for Cu and 98.3% for As in ERM-BB185, and between 84.2% for Co and 101.8% for Pb in NIST SRM 1640a. Following the quality-control checks performed every 12 samples, one previously analysed sample was selected at random and the same prepared solution was remeasured to check instrumental repeatability. Independent replicate digestions of the tissue samples were not performed. Values strictly below the element-specific limit of detection (LOD), including zeros, were replaced by LOD/√2 before calculating descriptive statistics, MPI and PCA. Below-LOD measurements occurred for Ni (7/73; 9.59%) and As (2/73; 2.74%); no below-LOD measurements occurred for the other 12 elements. Substitution retained all measured tissue samples, with fewer than 10% of observations censored for either affected element. Limits of detection for each element are provided in Supplementary Data. All tissue concentrations were expressed as mg/kg wet weight. Reported tissue concentrations are rounded to two decimal places for values ≥1 mg/kg, three for values from 0.01 to <1 mg/kg, and four for values <0.01 mg/kg; all calculations used unrounded values.

### 2.3. Metal Pollution Index

The Metal Pollution Index (MPI) was calculated as a composite indicator of overall metal(loid) accumulation in liver and kidney tissues. The index was computed using the following equation [19]:

MPI = (*Cf*_1_ × *Cf*_2_ …*Cf_n_)*^1/^*^n^*

where *Cf_i_* is the concentration of element i in the tissue sample, expressed as mg/kg, and *n* = 14. All analysed elements (As, Cd, Co, Cr, Cu, Fe, Hg, Mg, Mn, Mo, Ni, Pb, Se, and Zn) were included in the MPI calculation for each liver and kidney sample. The MPI was used to complement element-specific comparisons by summarizing the concentrations of multiple elements in a single descriptive measure. However, different elemental profiles may produce similar MPI values, so the index must be interpreted alongside the individual concentrations. The calculation does not weigh elements according to their essentiality or toxicological potency. Consequently, differences in MPI may reflect both variation in exposure and the physiological regulation of essential elements. A higher MPI indicates a higher geometric mean concentration of the included elements and should not be interpreted as evidence of greater mixture toxicity or adverse health effects.

### 2.4. Data Analysis

Statistical analyses were performed in R version 4.4.1. Descriptive statistics were calculated as medians together with minimum and maximum values. After LOD substitution, concentrations of the 14 elements were centred and scaled to unit standard deviation. A robust Principal Component Analysis (PCA) was performed using the Hubert method implemented in the rrcov package (version 1.7-7) to summarize multivariate variation in element concentrations. Four components were retained. All 73 available tissue samples from 41 individuals were included; missing organs were not imputed. Component loadings were visualized using a signed dot plot, and PCA scores were displayed in biplots generated with ggplot2 (version 4.0.3), including 95% normal-theory data ellipses for each species.

Linear mixed-effects models were then fitted to the Yeo–Johnson-transformed scores of the first four principal components, using bestNormalize (version 1.9.2) for transformation and lme4 (version 2.0-1) and lmerTest (version 3.2-1) for model fitting. Species, tissue, and their interaction were included as fixed effects, whereas individual was included as a random effect to account for paired liver and kidney measurements; individuals with only one available organ were retained. Overall fixed effects were evaluated with type III F-tests using Satterthwaite degrees of freedom. Individual coefficients were interpreted relative to the Egyptian mongoose and kidney as the reference levels. Model assumptions were evaluated by inspecting residual Q-Q plots and applying the Shapiro–Wilk test to residuals, while residual variance homogeneity was assessed using Levene’s test across species–tissue groups. Residual Q-Q plots are provided in Supplementary Figure S1.

### 2.5. Screening-Level Toxicological Risk Assessment

A screening-level toxicological risk assessment was performed to evaluate the potential biological relevance of metal(loid) concentrations measured in mesocarnivore tissues. The assessment used comparisons with published mammalian tissue-residue criteria expressed on a wet-weight basis and focused on the elements and organs for which suitable reference values were available.

For each individual and element–tissue combination with a suitable Toxicological Reference Value (TRV), a screening risk quotient (RQ) was calculated as:

RQ = *C*/TRV

where *C* is the measured tissue concentration (mg/kg) and TRV is the selected tissue-residue reference value (mg/kg). An RQ ≥ 1 denotes an exceedance of the selected reference value, rather than direct evidence of a toxic effect. Values below 1 indicate concentrations below that criterion, not the absence of every possible adverse effect.

For Cd, kidney concentrations were compared with the mammalian renal effect indicator of 30 mg/kg proposed by Environment Canada and Health Canada [20]. Because the same assessment reports renal tubular epithelial alterations in experimentally exposed mice at approximately 13–20 mg/kg, an exploratory sensitivity comparison identified individuals with Cd concentrations ≥13 mg/kg. This lower concentration was not treated as a validated threshold for the species examined.

For Hg, liver concentrations were compared with the 4.2 mg/kg boundary between the lowest (“No risk”, NRC) and next (“Low risk”, LRC) categories for terrestrial mammals proposed by Dietz et al. [21]. These categories are based on experimental findings in mink, and their applicability to the mesocarnivores investigated here is therefore indicative rather than species-specific.

Pb concentrations were examined descriptively to identify individuals with comparatively high burdens, but no formal RQs were calculated because of uncertainty in the applicability of available tissue-residue effect thresholds. Other elements were excluded from the quantitative assessment when sufficiently applicable tissue-based toxicological criteria could not be identified. For each species and tissue, medians, minima, maxima, maximum RQs and numbers of samples reaching or exceeding the selected reference values were calculated; the denominator for each percentage was the number of available tissue samples.

## 3. Results

### 3.1. Element Concentrations and Metal Pollution Index

Table 1 summarizes the median, minimum, and maximum concentrations of the analysed elements in kidney and liver tissues of Egyptian mongooses, stone martens, and Eurasian badgers. Mg, Fe, Zn, Cu, and Mn were generally among the most abundant elements across species and tissues. The highest Fe concentrations were observed in the liver of Eurasian badgers, with a median of 176.24 mg/kg and a range of 17.07–341.80 mg/kg. Cd concentrations in the kidney of Eurasian badgers were also higher than in the other species, with a median of 2.63 mg/kg and a maximum value of 17.35 mg/kg.

**Figure 2:**
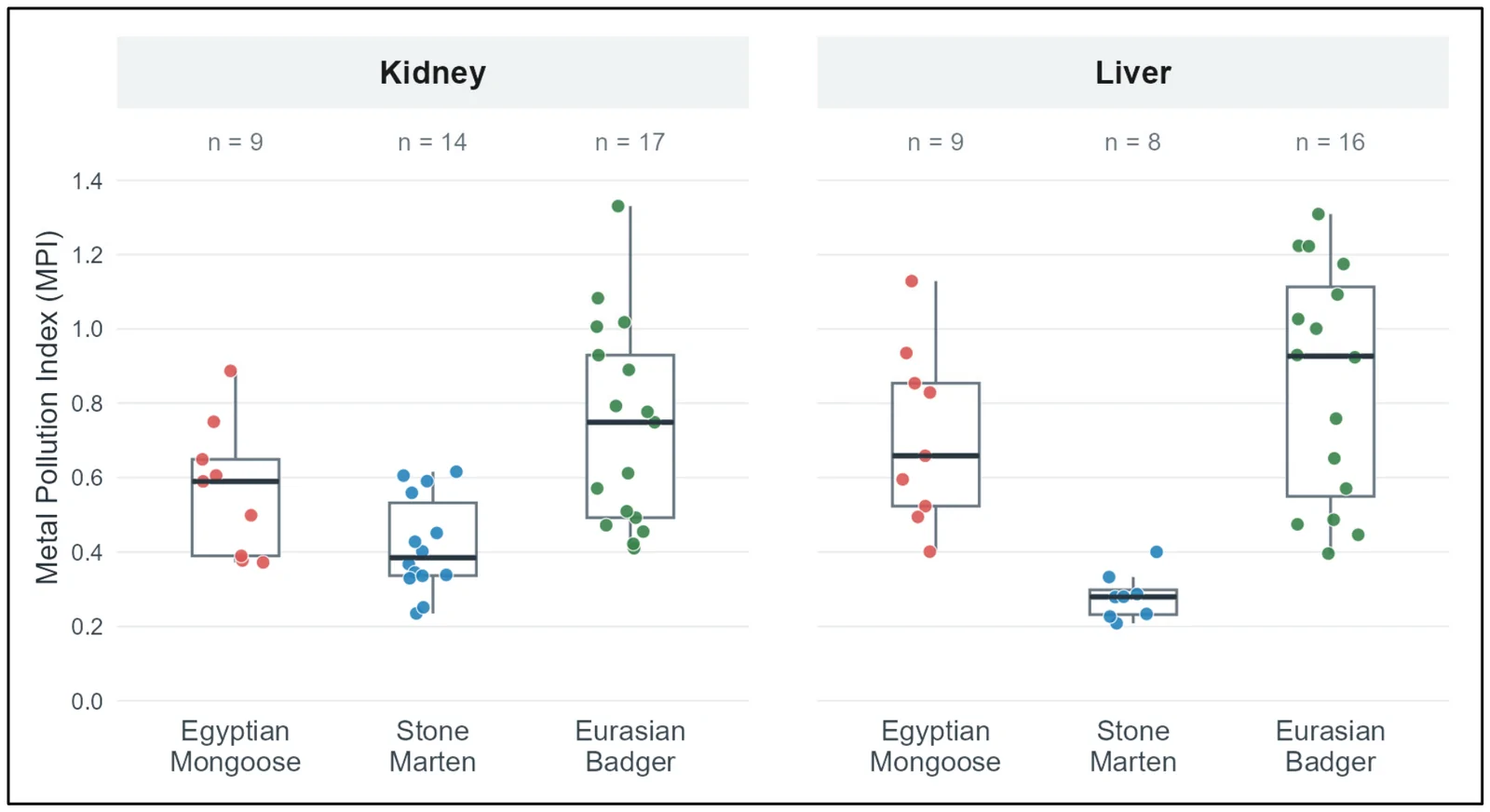
Metal Pollution Index (MPI) in kidney and liver samples from the three mesocarnivore species.

Figure 2 presents the MPI distributions for the three mesocarnivore species in kidney and liver. Boxplots summarize the median and interquartile range, while the overlaid points show the individual tissue samples. Eurasian badgers generally had higher MPI values in both tissues, stone martens had lower values, and Egyptian mongooses showed intermediate values.

### 3.2. Bioaccumulation Patterns in Mesocarnivores

**Figure 3:**
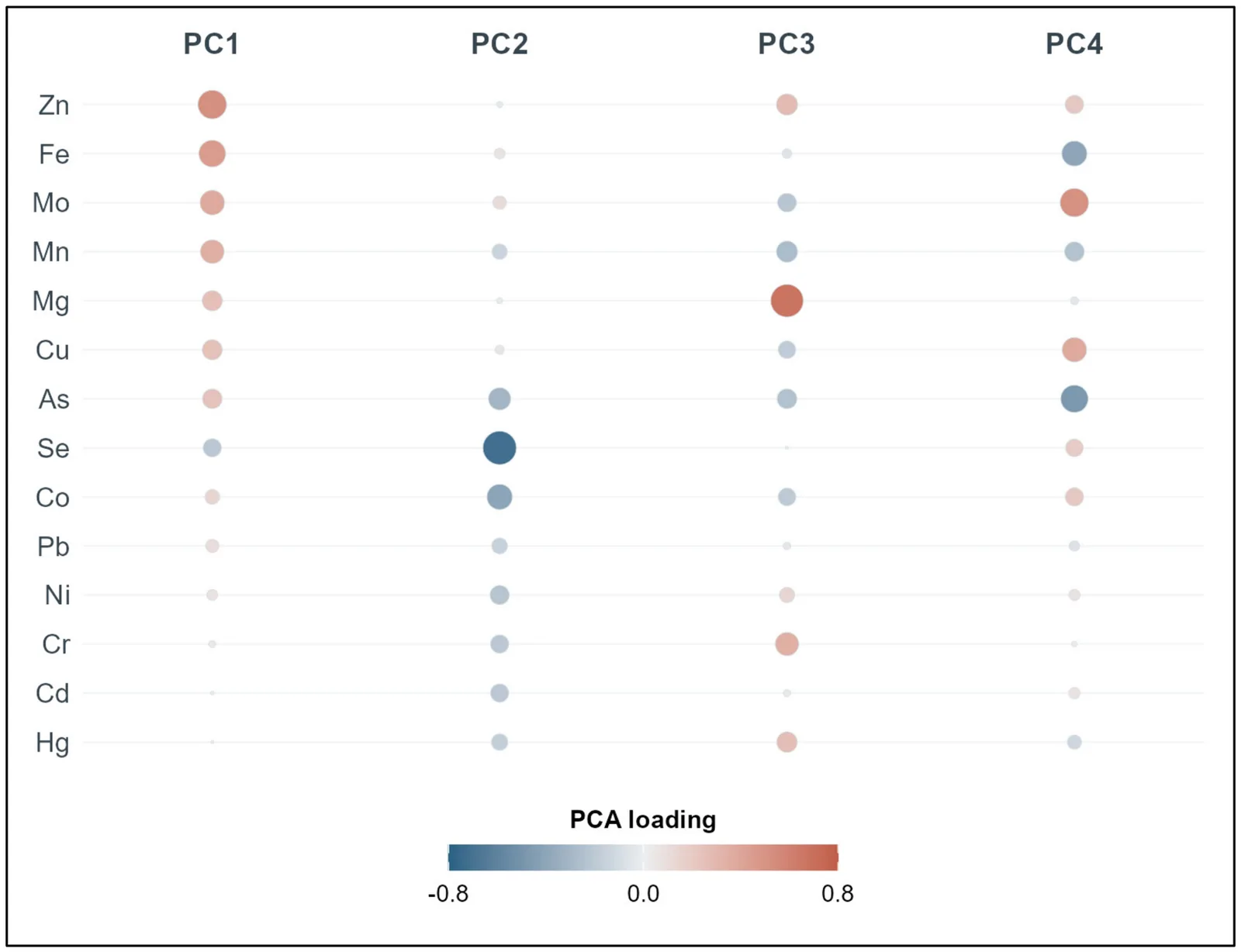
Loading plot of the analysed elements on the first four components of the robust principal component analysis. Circle area is proportional to the absolute loading; colour indicates its sign and magnitude.

The robust PCA identified four principal components that together explained 73.47% of the total variance. Specifically, PC1, PC2, PC3, and PC4 explained 36.99%, 19.54%, 8.75%, and 8.18% of the variance, respectively. Figure 3 displays the component loadings for each element. PC1 had its largest positive loadings for Zn, Fe, Mo, and Mn. PC2 was dominated by negative loadings of Se, Co, and As, with additional negative contributions from Ni, Cr, and Cd. PC3 was characterized by a strong positive loading for Mg, whereas PC4 showed positive loadings for Mo and Cu with negative loadings for As and Fe.

**Figure 4:**
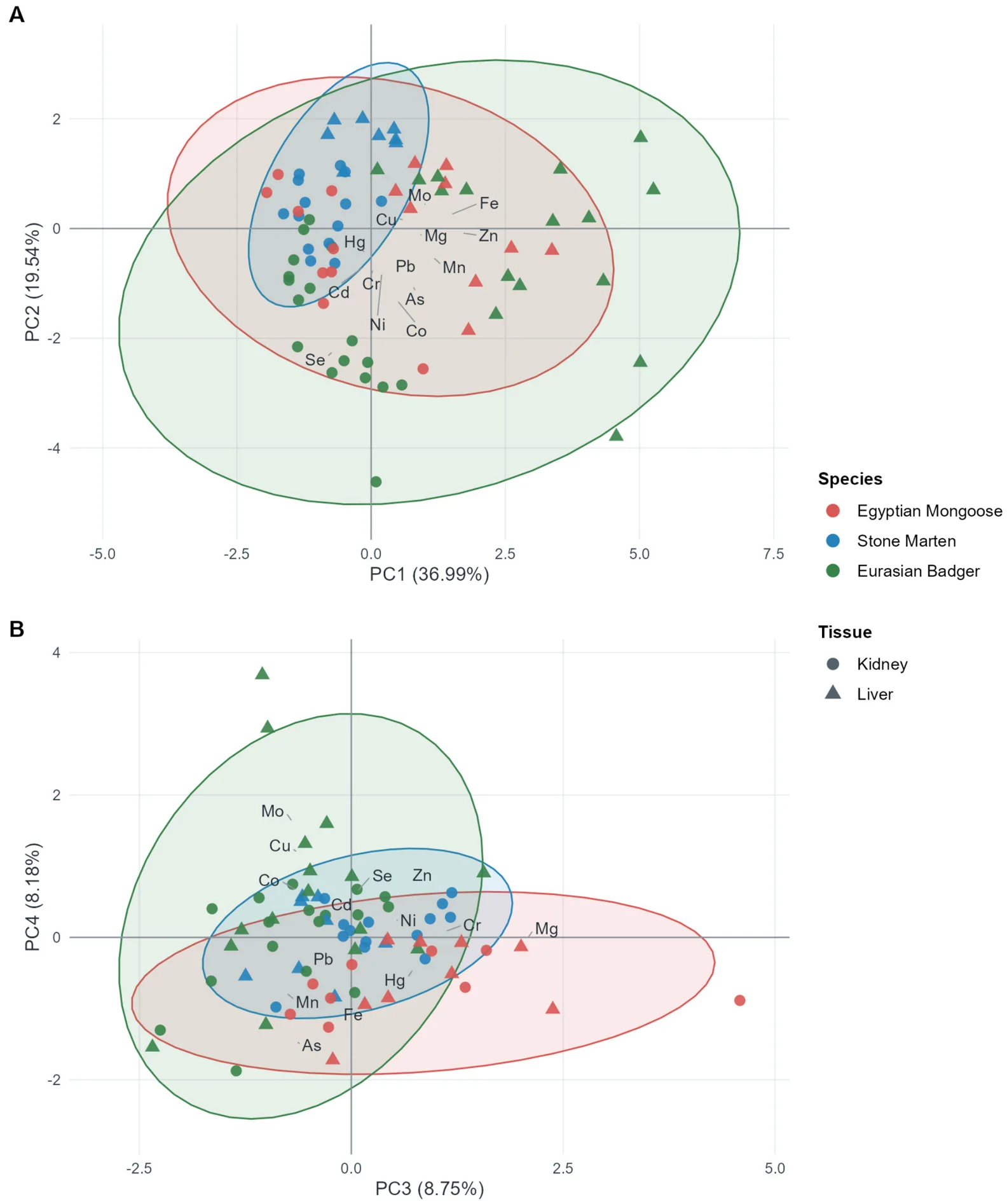
PCA biplots showing the distribution of species and tissues, together with the loadings of the analysed elements: (**A**) PC1–PC2 space; (**B**) PC3–PC4 space. Shaded areas represent 95% normal-theory data ellipses for each species. Circles denote kidney samples and triangles denote liver samples.

Figure 4 shows the distribution of species, tissues, and element loadings within the multivariate space defined by the principal components. Type III tests of the mixed-effects models identified effects of species (*p* = 0.001), tissue (*p* < 0.001), and their interaction (*p* = 0.002) on PC1. In the reference species, the Egyptian mongoose, liver scores were higher than kidney scores (coefficient *p* < 0.001). The liver–kidney difference was smaller in stone martens than in Egyptian mongooses (interaction coefficient *p* = 0.009).

For PC2, type III tests identified effects of species and tissue (both *p* < 0.001) and a species × tissue interaction (*p* = 0.006). Among kidney samples, Eurasian badgers had lower scores than Egyptian mongooses (coefficient *p* = 0.005), consistent with the negative loadings of several elements on this component. The liver–kidney contrasts differed from Egyptian mongooses in both stone martens (interaction coefficient *p* = 0.003) and Eurasian badgers (*p* = 0.006).

For PC3, species (*p* < 0.001) and the species × tissue interaction (*p* = 0.044) were significant in the type III tests, whereas the overall tissue effect was not (*p* = 0.765). Among kidney samples, Eurasian badgers had lower scores than Egyptian mongooses (coefficient *p* < 0.001); the liver–kidney contrast differed between stone martens and Egyptian mongooses (interaction coefficient *p* = 0.028). For PC4, the type III species effect was significant (*p* = 0.008), but neither the tissue effect (*p* = 0.085) nor the interaction (*p* = 0.075) was significant. Among kidney samples, stone martens and Eurasian badgers had higher scores than Egyptian mongooses (coefficient *p* = 0.014 and *p* = 0.042, respectively).

### 3.3. Screening-Level Toxicological Risk Assessment

The screening-level tissue-residue assessment revealed different patterns of potential toxicological concern for Cd and Hg across species (Table 2). None of the 40 renal Cd samples reached the principal reference value of 30 mg/kg. Eurasian badgers had the highest renal Cd concentrations (median, 2.63 mg/kg; maximum, 17.35 mg/kg), corresponding to a maximum RQ of 0.578. The maximum RQs for Egyptian mongooses and stone martens were 0.074 and 0.053, respectively.

Three of the 17 Eurasian badger kidney samples (17.6%) had Cd concentrations between 14.30 and 17.35 mg/kg, within the lower experimental effect range of 13–20 mg/kg reported in mice. These samples represented 7.5% of all kidney samples; none reached 20 mg/kg.

All 33 hepatic Hg concentrations were below 4.2 mg/kg. The maximum hepatic Hg concentration occurred in Egyptian mongooses (1.65 mg/kg; maximum RQ, 0.393), followed by Eurasian badgers (0.174 mg/kg; maximum RQ, 0.041) and stone martens (0.102 mg/kg; maximum RQ, 0.024). All individuals were therefore assigned to the lowest risk category (NRC) within the classification of Dietz et al. [21].

In the exploratory assessment of Pb, the highest concentrations were recorded in Eurasian badgers, reaching 3.18 mg/kg in liver and 2.29 mg/kg in kidney. No formal toxicity category was assigned for Pb. The screening assessment identified renal Cd accumulation in a subset of Eurasian badgers as the principal finding warranting further investigation.

## 4. Discussion

The concentrations measured in liver and kidney show that mesocarnivores living in and around a protected Mediterranean landscape are exposed to a range of metal(loid)s. The Sierra de Aracena area has a geochemical background influenced by weathering of sulfide-bearing deposits, which has been associated with elevated soil concentrations of Zn, Pb, Cd, and As [10]. This setting differs from nearby mining-affected areas, where anthropogenic inputs can make a substantial contribution to contamination [11]. Previous investigations in the park reported limited extractability of many soil-bound elements, whereas Cd and Zn were comparatively more available [22]. The relatively high renal Cd concentrations found in Eurasian badgers are compatible with this environmental context, although tissue measurements alone cannot establish the sources or routes of exposure.

Species-related differences were evident in the concentrations of several elements and in the MPI. Eurasian badgers generally had higher MPI values, stone martens had lower values, and Egyptian mongooses occupied an intermediate position. Feeding ecology may contribute to these differences. The diet of Eurasian badgers includes soil-associated invertebrates such as earthworms [23], which can accumulate metals and facilitate their transfer through terrestrial food webs [24,25]. Earthworms can acquire metals such as Cd and Zn through dermal uptake from soil pore water and through the ingestion of metal-bearing soil particles [26]. Earthworm consumption and incidental soil ingestion during foraging may therefore provide complementary routes of metal exposure for Eurasian badgers [27]. The consumption of plant material by stone martens [15] and the dietary flexibility of Egyptian mongooses [16,28] offer possible explanations for the patterns observed. These relationships remain hypotheses because diet and local prey contamination were not examined in the sampled animals. Moreover, the MPI represents the combined concentrations of essential and potentially toxic elements; a higher index therefore indicates greater measured metal(loid) accumulation, not necessarily greater toxicological risk.

Tissue-specific accumulation was also apparent. Liver samples generally contained higher concentrations of several essential elements, consistent with the metabolic and storage functions of this organ. Renal Cd was particularly prominent in Eurasian badgers. The affinity of Cd for metallothioneins and its retention in renal tissue provide a plausible mechanism for this pattern [29,30]. Although the toxicological relevance of Cd and Pb in mammals is well documented [6,31,32,33], tissue accumulation must be distinguished from evidence of adverse effects. The contrasting behaviour of essential elements and Cd also highlights the limitations of interpreting total metal(loid) burden without considering the individual elements and organs involved.

The robust principal component analysis complemented these comparisons by identifying covariation among elements and differences associated with species and tissue. PC1 was mainly related to Zn, Fe, Mo, and Mn and was associated with the distinction between liver and kidney samples. PC2 was dominated by negative loadings of Se, Co, and As, with Eurasian badger kidney samples tending towards lower scores than those of Egyptian mongooses. These loadings should not be interpreted as a single toxicological gradient, since the elements contributing to a component differ in their biological roles. The species-by-tissue interactions detected for PC1, PC2 and PC3 further suggest that accumulation patterns are not uniform across organs. The overlap among groups in the PCA plots indicates that the species and tissue profiles were not completely distinct. This combination of element-specific and multivariate information supports the potential value of wild mammals in exposure monitoring [34], while avoiding the assumption that a principal component score represents toxicological risk.

The screening-level assessment provided a more specific interpretation of the elements for which suitable tissue-residue reference values were available. Renal Cd concentrations remained below the selected reference value of 30 mg/kg wet weight [20]. Nevertheless, three of the 17 Eurasian badger kidney samples fell within the lower experimental effect range of 13–20 mg/kg reported in mice [20]. The observed concentrations cannot be used to infer renal injury in these badgers: that range is not a validated effect threshold for Eurasian badgers, and neither renal histopathology nor functional biomarkers were assessed. Cd accumulation has also been reported in wild badgers from the Iberian Peninsula [13], although differences in wet-weight and dry-weight reporting limit direct comparison. The finding points to renal Cd as a priority for studies that combine tissue residues with measures of biological effect, rather than demonstrating toxicity in the animals examined here.

All hepatic total Hg measurements were below the 4.2 mg/kg wet-weight boundary used to distinguish the lowest two categories in the assessment by Dietz et al. [21]. The highest hepatic Hg concentrations occurred in Egyptian mongooses, illustrating that the species with the greatest MPI was not necessarily the species with the greatest burden of every potentially toxic element. The Hg categories were derived largely from evidence in mink and should be applied to other mesocarnivores cautiously. Furthermore, only total Hg was measured; the absence of speciation data limits interpretation of the toxicological significance of the concentrations observed. In mammals, hepatic demethylation converts methylmercury (MeHg) to inorganic Hg, and species differ in their capacity to process and eliminate MeHg [35]. Consequently, interspecific differences in the relative proportions of MeHg and inorganic Hg may complicate comparisons based on total hepatic Hg; these proportions could not be assessed in the present study. Being below the selected reference value does not exclude effects that were not investigated in this study.

Pb concentrations showed considerable variation, especially in Eurasian badgers, but no risk quotients were calculated for Pb because an appropriate species- and tissue-specific effect criterion could not be established. Descriptive differences in Pb residues should therefore not be equated with differences in clinical risk. The use of tissue-residue screening values for Cd and Hg is likewise constrained by the extrapolation of experimental thresholds between mammalian species, although these comparisons can help identify elements and organs warranting further attention.

Roadkill sampling provides a practical means of assessing metal(loid) exposure in free-ranging mesocarnivores without additional animal capture [36,37]. In this study, it enabled comparison of three species and two target organs within a protected Mediterranean landscape. Interpretation should reflect the sampling design: road use and adjacent habitats, together with carcass detection, recovery and preservation, influence specimen availability, so the sample cannot be assumed to represent the wider populations. The small, unequal groups may limit statistical power and precision, particularly for species-by-tissue interactions; nonsignificant differences therefore do not establish equivalent exposure. Sex and body weight, recorded for 35 and 32 of the 41 individuals, respectively, were used descriptively because incomplete records and the sample size constrained their inclusion as model covariates.

Future studies can build on these comparisons by incorporating age and standardised body-condition data, which were unavailable here, and assessing diet and individual movements. Feasible age indicators, such as dental assessments, and body-condition measures should be recorded when carcass preservation permits reliable evaluation, with uncertain or unavailable observations documented. Complementary environmental sampling, assessment of local contaminant bioavailability, renal histopathology and functional biomarkers would help connect tissue residues with exposure pathways and biological effects. These endpoints were outside the scope of the present study, which describes tissue exposure and screening-level toxicological concern. Such work would be particularly valuable for interpreting renal Cd accumulation in Eurasian badgers and evaluating its implications for animal health and population-level risk.

## 5. Conclusions

This study shows that wild mesocarnivores can provide useful information on environmental metal(loid) exposure, even in protected areas where direct anthropogenic inputs may be limited. Clear differences in accumulation patterns were observed among species and tissues, highlighting the influence of both ecological traits and tissue-specific processes on metal(loid) burdens. Liver generally showed higher concentrations of essential elements, consistent with its central role in metabolism, storage, and detoxification, whereas the kidney was the main site of Cd accumulation, particularly in Eurasian badgers. In the screening-level assessment, none of the renal Cd or hepatic total Hg concentrations exceeded the selected reference values, although three badgers had renal Cd concentrations within a lower experimental effect range described in mice. This finding identifies a priority for further investigation, without demonstrating adverse biological effects. Eurasian badgers may be useful for monitoring prolonged exposure to soil-associated metals, particularly through renal Cd measurements. Higher hepatic Hg concentrations in Egyptian mongooses illustrate the complementary information provided by different species.

## Figures and Tables

**Table 1:** Median, minimum, and maximum concentrations of the analysed elements in kidney and liver tissues of the three mesocarnivore species.

Elements (mg/kg)	Species					
	Egyptian Mongoose		Stone Marten		Eurasian Badger	
	Kidney	Liver	Kidney	Liver	Kidney	Liver
As	0.036 (0.023–0.152)	0.057 (0.023–0.158)	0.0066 (<LOD–0.036)	0.0063 (<LOD–0.022)	0.048 (0.0086–0.254)	0.059 (0.020–0.312)
Cd	0.313 (0.142–2.21)	0.073 (0.029–0.415)	0.157 (0.0090–1.58)	0.025 (0.0025–0.101)	2.63 (0.050–17.35)	0.338 (0.0065–3.64)
Co	0.028 (0.010–0.070)	0.039 (0.011–0.060)	0.020 (0.011–0.038)	0.013 (0.0073–0.020)	0.048 (0.021–0.175)	0.062 (0.027–0.251)
Cr	0.0084 (0.0032–0.039)	0.0098 (0.0050–0.148)	0.010 (0.0046–0.034)	0.0085 (0.0033–0.025)	0.011 (0.0013–0.050)	0.010 (0.0026–0.103)
Cu	3.17 (2.68–7.21)	7.26 (3.72–19.81)	3.85 (2.96–4.88)	6.60 (5.21–16.66)	4.26 (2.67–8.49)	14.45 (2.43–68.29)
Fe	54.64 (6.60–70.40)	127.80 (84.00–176.96)	50.43 (33.23–76.55)	102.01 (17.80–154.09)	73.97 (25.73–136.69)	176.24 (17.07–341.80)
Hg	0.250 (0.114–0.980)	0.295 (0.138–1.65)	0.176 (0.044–0.344)	0.051 (0.018–0.102)	0.149 (0.058–0.549)	0.087 (0.021–0.174)
Mg	135.13 (108.64–342.20)	167.05 (153.65–189.16)	149.41 (120.61–183.41)	142.82 (115.37–158.10)	120.01 (93.28–177.22)	160.63 (122.03–214.72)
Mn	0.887 (0.455–3.74)	2.55 (2.01–9.37)	1.19 (0.664–10.43)	2.33 (1.19–5.31)	1.22 (0.643–7.84)	2.83 (1.55–13.20)
Mo	0.155 (0.101–0.204)	0.364 (0.236–0.439)	0.273 (0.170–0.512)	0.425 (0.205–0.572)	0.258 (0.145–0.349)	0.638 (0.378–0.927)
Ni	0.0090 (0.0013–0.150)	0.0092 (0.0007–0.044)	0.0072 (<LOD–0.048)	0.0023 (<LOD–0.034)	0.0077 (<LOD–0.041)	0.012 (<LOD–0.044)
Pb	0.123 (0.040–0.576)	0.147 (0.025–0.536)	0.054 (0.015–0.248)	0.025 (0.0070–0.049)	0.207 (0.029–2.29)	0.240 (0.025–3.18)
Se	1.04 (0.583–1.44)	0.678 (0.436–0.941)	1.15 (0.777–1.62)	0.377 (0.254–0.540)	1.88 (1.16–2.65)	0.744 (0.531–1.48)
Zn	20.17 (15.33–25.80)	38.42 (29.00–54.59)	22.15 (18.29–28.04)	22.06 (16.42–27.26)	22.25 (17.11–27.67)	38.57 (26.82–74.51)

Values are medians (minimum–maximum), in mg/kg wet weight. Sample sizes (kidney/liver): Egyptian mongoose, 9/9; stone marten, 14/8; Eurasian badger, 17/16. <LOD denotes a minimum below the detection limit (Ni, 0.0003 mg/kg; As, 0.001 mg/kg).

**Table 2:** Screening-level toxicological assessment of renal Cd and hepatic Hg concentrations in three mesocarnivore species.

Species	Element/Tissue	*n*	Median (Min–Max) mg/kg	TRV mg/kg	Max. RQ	*n* ≥ TRV
Eurasian Badger	Cd/Kidney	17	2.63 (0.050–17.35)	30	0.578	0
Stone Marten	Cd/Kidney	14	0.157 (0.0090–1.58)	30	0.053	0
Egyptian Mongoose	Cd/Kidney	9	0.313 (0.142–2.21)	30	0.074	0
Eurasian Badger	Hg/Liver	16	0.087 (0.021–0.174)	4.2	0.041	0
Stone Marten	Hg/Liver	8	0.051 (0.018–0.102)	4.2	0.024	0
Egyptian Mongoose	Hg/Liver	9	0.295 (0.138–1.65)	4.2	0.393	0

TRVs: renal Cd, 30 mg/kg [20]; hepatic Hg, 4.2 mg/kg [21].

## Data Availability

Data are contained within the Supplementary Data.

## Supplementary Materials

The following supporting information can be downloaded at: https://www.mdpi.com/article/10.3390/biology15212038/s1.
